# Connected at Sea: The Influence of the Internet and Online Communication on the Well-Being and Life Satisfaction of Cruise Ship Employees

**DOI:** 10.3390/ijerph17082840

**Published:** 2020-04-20

**Authors:** Aleksandar Radic, Antonio Ariza-Montes, Felipe Hernández-Perlines, Gabriele Giorgi

**Affiliations:** 1 Independent Researcher, 20000 Dubrovnik, Croatia; aleradic@gmail.com; 2 Management Department, Universidad Loyola Andalucía, 14004 Córdoba, Spain; 3 Facultad de Administración y Negocios, Universidad Autónoma de Chile, Santiago 425, Chile; 4 Department of Business Administration, University of Castilla-La Mancha, 45071 Toledo, Spain; felipe.HPerlines@uclm.es; 5 Department of Human Science, European University of Rome, 00163 Rome, Italy; prof.gabriele.giorgi@gmail.com

**Keywords:** online communication, well-being, life satisfaction, cruise ship employees, social support, social pressure, fear of missing

## Abstract

This study aims to elucidate the idiosyncratic effects of the Internet and online communication on the well-being and life satisfaction of cruise ship employees. Cross-sectional surveys and covariance-based structural equation modelling tools were used. In addition, univariate variance analysis was used to address the effects of socio-demographic variables (years of service on a cruise ship, working department on a cruise ship, gender, age, educational level and place of residency) on latent variables of the conceptual model. The conceptual model draws on existing theory and previous research and was empirically tested on a sample of cruise ship employee internet users. Result show that while being onboard a cruise ship, employees experience strong social pressure to be constantly available and they fear of missing out on important information and life events. Thus, relatedness to friends and family needs satisfaction is of paramount importance for cruise ship employees because they are fully aware that they are dispensable and replaceable to cruise ship companies, however to their friends and family, they are indispensable and unique. Moreover, employees who engage in other tasks/activities while taking part in online communication with friends and family exhibit reduced performance, which leads to poor interaction and social dissatisfaction. Lastly, employees experiencing under-reciprocating exchanges show significant negative effects on their well-being. Overall, the results provided several important theoretical and practical implications relevant to cruise tourism and human resource management.

## 1. Introduction

Cruise tourism growth for 2020 predicted by [1] will not be achieved due to the recent cruise tourism crisis caused by the COVID 19 pandemic. On 14 March 2020, all cruise lines suspended their cruise operations for at least 30 days [2]. However, none of the cruise companies have filed for bankruptcy or cancelled their new builds. Therefore, the prediction of the addition of 80,000 new crew members and officers each year until 2027 remains an achievable possibility [3]. A recent study by [4] revealed that cruise ship employees are exposed to long working hours and detachment from friends and family, which leads to poor social interactions and feelings of loneliness. However, advances in information and communication technologies have led to their widespread and increased usage by employees. Internet access has become a basic necessity, a more essential element of their mundane ship life with paramount importance on their well-being and life satisfaction. Enhanced connectivity is instrumental for bolstering morale and reinforcing job satisfaction, which ultimately strengthens the capability for communication between employees and their significant others back home and hence, reduces the feeling of loneliness [5]. Moreover, Maritime Labour Convention [6] recommended rational access to the Internet with reasonable charges for services. From November 2019 to February 2020, only a handful of cruise companies (Disney Cruise Line, Holland America, Azamara, and Princess Cruises) have provided free-of-charge specialized cross-platform messaging Internet applications for their employees. Interestingly, the Seafarers Happiness Index, which covers 10 aspects of job quality, including mental and physical health and relationships at home and onboard, showed significant increases in happiness for cruise ship employees from 5.3 to 7 out of 10 [7,8]. 

Given the unique work and life conditions on cruise ships, whereby employees are set apart from their loved ones [9], free Internet access should be a universal entitlement [10] because of its ability to enhance seafarer morale, engagement, well-being and life satisfaction [11]. Although significant amounts of research have been done on the positive effects of Internet and online communication on social pressure [12], fear of missing out [13], relatedness to friends and family need satisfaction [14], perceived social support [15], well-being [16] and life satisfaction [17], these effects were never studied in the peculiar environment of a cruise ship where life and work contexts are so intertwined such that the distinction between one and the other is blurred [18].

This study aimed to elucidate the idiosyncratic effects of the Internet and online communication on the well-being and life satisfaction of cruise ship employees. We reviewed existing theory and previous studies on the effects of the Internet and online communication on social pressure, fear of missing out, Internet multitasking and relatedness to friends and family need satisfaction. We investigated the influence of social pressure and fear of missing out on relatedness to friends and family need satisfaction and Internet multitasking. Finally, we proposed relatedness to friends and family need satisfaction and Internet multitasking as possible catalyst influencers of perceived social support which, in the end, are the impetus towards the well-being and life satisfaction of employees. The conceptual model draws on existing theory and previous research and was empirically tested on a sample of employee internet users. Finally, we addressed the effects of socio-demographic variables (years of service on a cruise ship, working department on a cruise ship, gender, age, educational level and place of residency) on latent variables of the conceptual model. This study is exploratory in nature and presents work addressing a major research gap, given that the effects of Internet and online communication on the well-being and life satisfaction of cruise ship employees have never been empirically tested. The results of this study will contribute towards the further development of cruise tourism theory and strengthen existing theories, such as Theory of Belongingness [19], Self-Determination Theory [20], Uses and Gratification Theory [21], Conservation of Resources Theory [22] and the paradigm of Positive Psychology [23].

## 2. Literature Review and Hypothesis Development

### 2.1. Internet and Online Communication as a Driver of Social Pressure, Fear of Missing out and Relatedness to Friends and Family Need Satisfaction

The recent advancements in communication technologies have altered how human beings communicate and associate with one another [24]. The belief that free Internet is a moral human right [10] and should be provided to all seafarers, including cruise ship employees [5] has become increasingly popular. Internet access enhances our lives by providing free, instant, worldwide communication [10]; social network sites play central roles in our everyday activities [25] due to their capability to extend and connect social capital that has stimulating effects on personal psychological well-being [26]. Internet and online communication were introduced as a vision of a connected world where anybody could share experiences and feel less alone [27]. 

While working and living on cruise ships, employees are detached from their family and friends [9]. Primary communication instruments include Internet and online communications [5], which play significant roles in integrating work and family domains [28]. These communication instruments provide social capital, information and wider perspectives [25]. In recent survey conduct by [8], the authors concluded that onboard crew members experience strong social pressure for being constantly available to their family and friends. Today, almost every cruise ship employee has a mobile device [11]. Although mobile devices allow users to be constantly available, they also create an environment that increases social pressure [29]. Moreover, the social pressure to be constantly available is strongly related to communication load [13], with a suppressing effect on well-being via social overload [30]. Based on the social norm of reciprocity in friendship and family ties, psychological tensions and social pressures may arise [16]. Thus, based on the literature review and empirical findings, the following hypothesis is proposed:

**Hypothesis 1** **(H1).** *There is a positive relationship between the Internet and online communication and social pressure.*

In their Theory of Belongingness, Baumeister et al. [19] argue how human beings have an irresistible need to be a part of a group. The hardest part for cruise ship employees is being away from home and missing so many important life events and quality time with family and friends [18]. Similar results have been reported by [5] who demonstrated in their comprehensive report how due to being uncontactable at sea, seafarers miss key life events. Cruise ship employees are fully aware that work-life on a cruise ship comes with a great burden [31]. However, they are not willing to tolerate any lack of connectivity [8]. Internet and online communication are closely linked to fear of missing out [32]. Fear of missing out is defined as “a pervasive apprehension that others might be having rewarding experiences from which one is absent” [33]. Thus, we can conclude based on Compensatory Internet Use Theory [34] that individuals who feel that their life needs are not fulfilled and that they are missing important life events and social motivation will experience strong stimulation to use online communication and social networking sites. Based on the theoretical background, literature review and empirical findings, the following hypothesis was derived:

**Hypothesis 2** **(H2).** *There is a positive relationship between the Internet and online communication and fear of missing out.*

The Internet, social networking sites and online communication have become our liaisons, special amusers, Cerberus of our memories and, in times of need, even our counsellors [27]. Based on Self-Determination Theory [20], relatedness to friends and family need satisfaction is one of three basic psychological needs that foster healthy self-regulation and promote mental and physical health. Opportunities to experience positive feelings of proximity and affection with family and friends at home through the use of online communications benefits seafarers and their friends and family [5]. Interestingly, in the latest survey conducted by [11], although provisions of internet access for personal use had positively affected seafarer mental health and morale, home-related anxieties have remained the same, despite speculation that increased communications with family might generate more anxieties. The effects of the Internet and online communication and relatedness to friends and family need satisfaction remain unclear [35]. Previous studies have shown that internet and online communication are mainly linked to positive outcomes of relatedness to friends and family need satisfaction [36,37]. Few studies have demonstrated the opposite [25,38]. Thus, based on literature review, theory and empirical findings, we put forward the following hypothesis:

**Hypothesis 3** **(H3).** *There is a positive relationship between the Internet and online communication and relatedness to friends and family need satisfaction.*

### 2.2. Social Pressure and Fear of Missing out as Stimulus to Relatedness to Friends and Family Need Satisfaction and Internet Multitasking

Social pressure is conformist behaviour with multiple determinations when an individual or group craves specific social attention; this leads them to behave in certain ways unconcerned of any prestige advantage [39]. A recent study conducted by [13] showed how computer-meditated communication is portrayed and influenced by robust rules of conduct, where communication arrangements are under constant social pressure. Interestingly, within seafarers, social pressure has shown a strong influence on their work-life at sea due to their social isolation, imbalanced family life, separation from home, family and friends, and lack of free onboard communication facilities [40]. Moreover, relatedness proposes that cruise ship employees need to feel connected with their family and friends at home [5,9,18]: when employees feel satisfied with this need, they experience higher levels of work engagement and well-being. Most employees own smartphones and experience social pressure to make themselves available to friends and family at home, thereby satisfying needs for relatedness. Online communications induced by social pressure and relatedness to friends and family needs satisfaction has been directly linked with significant effects on user life satisfaction [41]. Thus, based on the literature review, theoretical background and empirical findings, we put forward the following hypothesis:

**Hypothesis 4** **(H4).** *There is a positive relationship between social pressure and relatedness to friends and family need satisfaction.*

The constant occupation with smartphones has created a peculiar mindset in users with specific feelings of being permanently online and connected [42]. Interestingly, online communication and social media are closely linked to fear of missing out [13]; 56% of U.S. social media users suffer from fear of missing out [43]. Thus, tensions related to social relationships may be the prevailing cause for the fear of missing out [44]. The Uses and Gratifications Theory provides us with pragmatic theoretical lenses instrumental in understanding underlying motives and multitasking behaviours [45]. The theory proposes that hidden roots of social and psychological needs create certain expectations from various media, which guides users towards specific models of media exposure, culminating in need gratification [46]. Looking at the advantages of online communications to those living and working at sea, fear of missing out has a significant impact; 75% of crew members need to be connected with the outside world, and 63% would leave their current company to join some other that would provide better onboard connectivity [5]. Previous research also showed that higher levels of fear of missing out had an impact on a higher tendency to internet multitask [33]. Cruise ship employees have long working hours and rarely go ashore; the tendency to internet multitask leads to some occupational injuries [4]. Consequently, based on theory, literature review and empirical findings, the following hypothesis is derived:

**Hypothesis 5** **(H5).** *There is a positive relationship between fear of missing out and internet multitasking.*

### 2.3. Relatedness to Friends and Family Need Satisfaction and Internet Multitasking as a Catalyst of Perceived Social Support

Widespread adoption of the Internet, online communication, and social network sites have empowered people across the globe to grow their social network [47]. However, such a gift comes with responsibility because various social network sites compete for our attention by streaming content based on well-programmed algorithms founded on our likes, fears, and needs [27]. The digital world is doused with ever-growing social network sites that are changing the online behaviour of digital technology users but also how human beings interact with one another in real life [48]. Moreover, human beings are social animals [49] in need of relatedness to friends and family, regardless of recent technological advancements. Relatedness comes in the form of affective needs, which tend to intensify delightful and affecting experiences, and social needs that tend to bolster existing connections with family and friends [50]. Thus, the need for relatedness is a feeling of satisfaction that comes from being a connected part of a community where individuals manifest a willingness to care about each other [51]. Being away from their homes in an isolated environment [52], cruise ship employees are constantly looking for social support [53]. Interestingly, perceived social support, characterized as the tangible or intangible support received from an individuals’ social circle, is associated with superior life satisfaction [54]. Consequently, based on theory, literature review and empirical findings, the following hypothesis is derived:

**Hypothesis 6** **(H6).** *There is a positive relationship between relatedness to friends and family need satisfaction and perceived social support.*

The term internet multitasking refers to “any combination of internet use with other media or non-media activities” [13] p. 94. The main reasons for multitasking are social interactions with friends and family and information seeking [45]. Cruise ship employees are most often in different time zones than their friends and family. This leaves them with a limited time frame for online communication and significantly lowers the opportunities for giving and receiving much needed social support. Moreover, cruise ship employees are under extreme time pressure due to long working hours [4]; this is when individuals perceive that if they engage in internet multitasking, they would be efficient [45]. However, the effects of internet multitasking on retention of information during online messaging and cognitive load, showed significant retention loss among simultaneous multitasks [55]. Similarly, internet multitasking has been associated with lower gratification and perception memory achievement and sensitivity and moderate standard bias [56]. Moreover, different types of multitasking have robust effects on task performance. Task performance significantly decreased when the given task was a secondary task, when a neurological obstruction was high, and when the behavioural reaction was present [57]. Thus, based on literature review and taking in consideration the conflicting empirical results of Internet multitasking effect on perceived social support, and bearing in mind the importance of perceived social support for cruise ship employees, the following hypothesis is derived:

**Hypothesis 7** **(H7).** *There is a positive relationship between internet multitasking and perceived social support.*

### 2.4. Perceived Social Support as a Catalyst towards Well-Being and Life Satisfaction

The social relationship is flexible and essential for individual vitality because human beings exist within larger social contexts where friends and family play important roles [58]. Moreover, if a persons’ social context is supportive of significant relationships, then these individuals encounter elevated feelings towards psychological needs, which can be satisfied through social synergy [41]. Social support has four dominant aspects in the creation of well-being and life satisfaction: main effect (adding particular supplementary function to mental health), mediating effect (intervening in relations between its precursors and health results), indirect effect (preventing disorders by framing mental health) and moderating effect (lowering the risk of any mental health-related components) [59]. Previous studies have shown that the Internet and online communication have a positive effect on social capital [60] where social capital is an antecedent of social support [59]. Internet and online communication are bonding and bridging social capital [38], which are of paramount importance for satisfying the social support need of cruise ship employees who tend to use online communication to contact geographically dispersed close friends and family [5]. Thus, social support plays multiple roles in individuals’ well-being and life satisfaction [59]. Considering the importance of well-being and life satisfaction of employees, based on literature review and empirical findings, we put forward the following hypotheses:

**Hypothesis 8** **(H8).** *There is a positive relationship between perceived social support and well-being.*

**Hypothesis 9** **(H9).** *There is a positive relationship between perceived social support and life satisfaction.*

People should focus on how to be happy, satisfied and filled with positivity [23]. Thus, psychological well-being is an essential part of positive psychology. Psychological well-being is related to ones’ feelings and evaluations about their life [61]. Moreover, well-being is seen as a psychological well-being that develops based on the eudaimonic dimension of well-being [62] and as happiness that is built around life satisfaction based on the hedonic dimension of well-being [63]. In the context of cruise ship employees, well-being is a fusion of eudaimonic (efficiency) and hedonic (thrill) dimensions. Interestingly, Gibson et al. [64] argues how due to work-life time constraints, task assignments and job anxiety, employees experience poor well-being. Moreover, Radić [18] questions the life satisfaction and well-being of employees who are economic gladiators in pursuit of an unobtainable economic freedom. Thus, Moore [65] calls cruise ships “misery machines” where in recent years, as Walker [66,67] point out, there has been a substantial increase in suicide rates due to the poor well-being of employees. Interestingly, perceived social support from online communication has had a positive effect on well-being [5,8]. Social network sites have provided ambient awareness that increases the well-being of its users [68].

**Figure 1:**
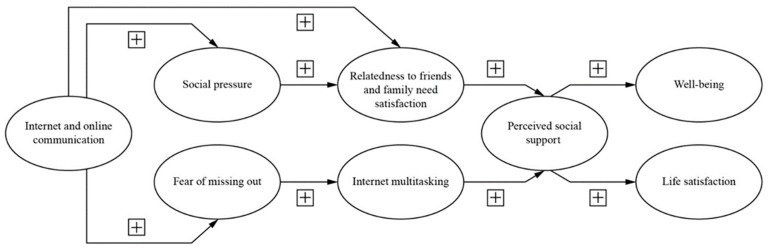
Research model and hypotheses.

Life satisfaction is related to a subjective, comprehensive evaluation of one’s quality of life [69]. Moreover, life satisfaction draws from the individual’s psychological aspects and is related to one’s hedonic satisfaction [62], where at the same time, perceived social support from an individual’s social networks has the potential to strengthen a person’s life satisfaction [24]. In the Q4/2019 report, Seafarers Happiness Index [8] showed how free online communications have a significant impact on employee life satisfaction; as Ang et al. [41] argue, computer-mediated communications can enhance life satisfaction. Online communication and social network sites can drive a person towards achieving superior life satisfaction and better quality social relationships [38]. Interestingly, although employees use online communication and social network sites to strengthen their close interpersonal connections and enhance their life satisfaction [18,70] argue that weak ties are also valuable due to their potential to positively influence life satisfaction. Figure 1 illustrates the research model and hypotheses of this study.

## 3. Methodology

### 3.1. Research Model, Design and Participants

The theoretical framework of this study was based on a literature review; the conceptual model and hypotheses were tested based on a convenience sample. The post-positivistic paradigm was adopted in this study because as [71] argues, the post-positivistic paradigm takes into consideration the fact that in human behaviour studies, observations are imperfect with potential inaccuracies; thus, all theories could be amended. Action research strategy allows the research to use different models of contemporary knowledge in solving genuine industry issues and applying obtained results outside the boundaries of the study [72]. Thus, action research strategy was used. The research model was evaluated using a cross-sectional survey and covariance-based structural equation modelling (CB-SEM). CB-SEM allows testing and validation of current theories and comparisons of different theories [73]. In summary, this study used a deductive approach followed by a cross-sectional time horizon and quantitative techniques for data collection. 

A comprehensive self-reported online survey in English was designed at SurveyMonkey®. Possible participants were invited to take part in the survey via Facebook group “Crew Center”. The main criteria was that participants had to be onboard and employed by a cruise company. The survey was online from 24 August to 1 December 2019, and the final sample consisted of 532 cruise ship employees (see Table 1). The sample comprised 328 males and 195 females from different geographical areas (43.8% from Europe, 20.3% from North America, 16.4% from Southeast Asia, 7.3% from South America, 4.6% from Central America, 4.2% from Africa, and 3.4% from Australia). Most respondents were between 31–40 years old (50.9%) followed by respondents 21–30 years old (27.7%) and 41–50 years old (11.9%). Among the participants, 49.5% were employed in the hotel department, 32.9% were from the deck and technical department, and 17.6% were from the entertainment department. Most (45.9%) had worked in the industry for over six years. A large share of respondents had a bachelor’s degree (57.7%). This extreme unrepresentative value was related to the convenience sampling method. Overall, the sample was a very good representation of employee demographics [74].

### 3.2. Measures

#### 3.2.1. Internet and Online Communication

Internet and online communication were assessed using a five item scale designed to asses internet and online communication usage (all the measures are included in the Appendix A). Participants indicated on a five point scale from 1 (once per week) to 5 (several times per week) how often they use the Internet for communication; from 1 (less than an hour) to 5 (more than 4 hours per day) how many hours per day (on average) they spend on internet communication; from 1 (once a week) to 5 (several times per day) how often they use a) instant messenger, b) social networking sites, and c) chat rooms. Internal consistency in the present sample was acceptable (Cronbach’s α = 0.790). 

#### 3.2.2. Social Pressure

Social pressure to be permanently available was assessed with an adapted perceived norm scale [13] that had four items (e.g., "People from my private social environment think that it is important that I’m constantly available") and is rated on a five point scale ranging from 1 (does not apply at all) to 5 (fully applies). Internal consistency in the present sample was acceptable (Cronbach’s α = 0.794). 

#### 3.2.3. Fear of Missing out

Fear of missing out on important life events and information was assessed with a three item scale (e.g., "If I would use the Internet less frequently, I would be missing out on important things") developed by [13]. Participants rated the items on a five point scale ranging from 1 (does not apply at all) to 5 (fully applies). Internal consistency in the present sample was good (Cronbach’s α = 0.845). 

#### 3.2.4. Relatedness to Friends and Family Need Satisfaction 

Relatedness to friends and family need satisfaction was assessed with three item scale (e.g., “I feel that my friends and/or family sincerely care about me”) developed by [75]. Participants rated the items on a five point scale ranging from 1 (strongly disagree) to 5 (strongly agree). Internal consistency in the present sample was good (Cronbach’s α = 0.905). 

#### 3.2.5. Internet Multitasking

Internet multitasking was assessed with a five item scale (e.g., “How often do you use the Internet while you simultaneously are in a conversation with another person”) developed by [13]. Participants rated the items on a five point scale ranging from 0 (never) to 4 (very frequently). Internal consistency in the present sample was acceptable (Cronbach’s α = 0.747). 

#### 3.2.6. Perceived Social Support

Perceived social support was assessed by the multidimensional scale of perceived social support [76], which consisted of six items (e.g., “There is a special person who is around when I am in need”) and was rated by participants on a seven point scale ranging from 1 (strongly disagree) to 7 (strongly agree). Internal consistency in the present sample was acceptable (Cronbach’s α = 0.784). 

#### 3.2.7. Well-Being

Well-being was assessed by The World Health Organization Well-Being Index [77]. It comprises five items (e.g., “I have felt cheerful and in good spirits”) and was rated by participants on a six point scale ranging from 1 (all the time) to 6 (at no time). Internal consistency in the present sample was good (Cronbach’s α = 0.875).

#### 3.2.8. Life Satisfaction

Life satisfaction was assessed by satisfaction with life scale [78]. It consists of five items (e.g., “In most ways my life is close to my ideal”) and it was rated by participants on a seven point scale ranging from 1 (strongly disagree) to 7 (strongly agree). Internal consistency in the present sample was good (Cronbach’s α = 0.869).

### 3.3. Data Analytic Procedure

Structural equation modelling (SEM) was computed using the AMOS 21 software packet (IBM, Chicago, Illinois), and the maximum likelihood method was used to estimate the parameters from the conceptual model (see Figure 1). The Kolmogorov-Smirnov and Shapiro-Wilks test showed that none of the variables were normally distributed. Thus, a Maximum Likelihood (ML) estimator with enough resistance capabilities to none-extreme deviations from the normal distribution [79] was used. Model fit was tested based on the χ2 and CMIN/df statistics, the comparative fit index (CFI) and the root mean square error of approximation (RMSEA) as recommended by [80]. The univariate analysis of variances (ANOVA) was used in search of differences among employee demographics and conceptual model variables. 

### 3.4. Results

#### 3.4.1. Results of the Structural Model

The model showed an acceptable fit to the data with the following values: χ2(483) = 1517,145, p = 0.000; RMSEA = 0.064, LO 90 = 0.060, HI 90 = 0.068; CMIN/DF = 3.141 and CFI = 0.887. Although the general indicator χ2 was significant, with such a large number of degrees of freedom, χ2 is not reliable; it is better to rely on other indicators. RMSEA was close to the limit that indicates an excellent model (0.06), CMIN/DF was within the limits that represent a good model, whereas the CFI was close to the lower limit of acceptability of the model [80,81,82]. Table 2 shows the hypothesized paths of the conceptual model. 

**Figure 2:**
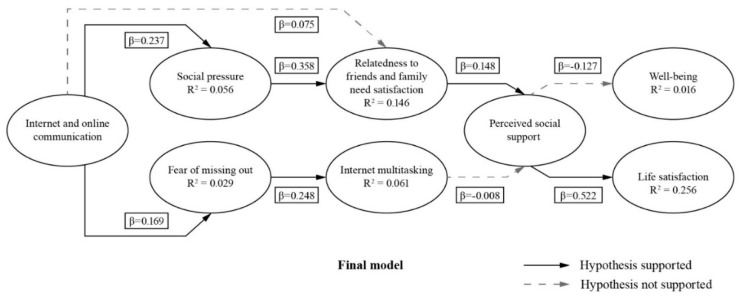
Hypotheses (supported and not supported).

The zero-order correlations between social pressure and fear of missing out, demonstrate that these two variables are strongly interrelated. Social pressure and fear of missing out show very high correlations (r = 0.79, p < 0.01). This significant relationship is reasonable: social pressure as a concept is closely connected to the concept of fear of missing out as these social processes on the Internet and social network sites are synthesized. Because social interaction through the Internet and online communication are of paramount importance for cruise ship employees [5], accomplishing this pursuit is a way towards well-being and life satisfaction. This conception is supported by results from [7,8], who reported that a recently developed free-of-charge specialized cross-platform messaging Internet application (by a handful of cruise companies) had an immediate impact on the happiness index of employees by increasing to 32% from Q2/2019 to Q4/2019. Except for hypotheses 3, 7 and 8, all other hypothesized relationships were supported in the final model (Figure 2).

Hypotheses 1 and 2 were supported showing how the Internet and online communication had positive effects on social pressure (β = 0.169) and fear of missing out (β = 0.237). Interestingly, Hypothesis 3 was not supported, demonstrating that the Internet and online communication did not have a positive effect on relatedness to friends and family need satisfaction (β = 0.075). As predicted in Hypothesis 4, social pressure had a positive effect on relatedness to friends and family need satisfaction (β = 0.358), and fear of missing out has a positive effect on internet multitasking (β = 0.248) as predicted in and Hypothesis 5. Hypothesis 6 predicted that relatedness to friends and family need satisfaction had a positive effect on perceived social support; this hypothesis was supported (β = 0.148). Hypothesis 7 predicted that internet multitasking had a positive effect on perceived social support; however, this hypothesis was not supported (β = −0.008). Hypothesis 8 predicted that perceived social support had a positive effect on well-being; however, this hypothesis was not supported (β = −0.127). Lastly, hypothesis 8 predicted that perceived social support had a positive effect on life satisfaction; this hypothesis was supported (β = 0.522).

#### 3.4.2. Sample Characteristics and Research Model

In pursuit of elucidating the peculiar socio-demographics characteristics of cruise ship employees and unrevealing significant differences on sample and research model variables, ANOVA was used. The results disclosed the following pivotal differences.

The number of years of service affected the research model variables: relatedness to friends and family need satisfaction (F(2, 296.23) = 7.49, p = 0.00), life satisfaction (F(2, 254.04) = 13.39, p = 0.00) and well-being (F(2, 520) = 17.19, p = 0.00). Tukey’s honestly significant difference (HSD) showed that employees with 6+ years of service showed significant differences (M = 4.03, SD = 0.76) towards relatedness to friends and family need satisfaction when compared with employees with between 3–5 years (M = 4.24, SD = 0.53) and less than 2 years of service (M = 4.28 SD = 0.53). Similarly, employees with 6+ years of service demonstrated momentous differences (M = 4.39, SD = 0.81) concerning life satisfaction when compared with employees with between 3–5 years (M = 4.06, SD = 0.98) and less than 2 years of service M = 3.89, SD = 1.01). In line with previous findings, employees with 6+ years of service had important differences (M = 1.90, SD = 0.83) with well-being and life satisfaction when compared with employees with between 3–5 years (M = 2.15, SD = 0.91) and less than 2 years of service (M = 2.50, SD = 0.96). Interestingly, employees who work on a cruise ship between 3–5 years showed noteworthy differences (M = 2.15, SD = 0.91) when compared with employees who worked less than 2 years (M = 2.50, SD = 0.96).

A cruise ship has various working departments; thus, interesting differences were observed in the research model variables among cruise ship employees of different working departments. The type of working department had a noticeable effect on relatedness to friends and family need satisfaction (F(2, 289.88) = 10.35, p = 0.00). Tukey’s HSD revealed that employees who worked in the Entertainment department showed significant differences (M = 4.36, SD = 0.43) towards relatedness to friends and family need satisfaction when compared with employees in the Hotel (M = 4.16, SD = 0.72) and Marine and Technical (M = 4.06, SD = 0.64) departments. 

Cruise companies support gender equality. To understand any remarkable differences between cruise ship employee gender and research model variables, a Student’s t-test was used. The results indicated that employee gender had significant effects on life satisfaction (t(521) = −2.99, p = 0.00) and well-being (t(521) = 2.81, p = 0.01). Interestingly, men (M = 4.27, SD = 0.94) exhibited slightly more life satisfaction than women (M = 4.02, SD = 0.90). However, women exhibited slightly more well-being (M = 2.25, SD = 0.95) than men (M = 2.02, SD = 0.87).

Age groups also had effects on relatedness to friends and family need satisfaction (F(4, 82.06) = 3.05, p = 0.02), well-being (F(4, 518) = 5.25, p = 0.00) and life satisfaction (F(4, 518) = 2.99, p = 0.02). Differences among age groups were low for relatedness to friends and family need satisfaction (η2 = 0.03) and life satisfaction (η2 = 0.02) and moderate for well-being (η2 = 0.04). Going forward, Student’s t-test revealed that employees between 21–30 years showed significant differences (M = 4.29, SD = 0.50) towards relatedness to friends and family need satisfaction when compared with those 41–50 years (M = 3.99, SD = 0.88) and 51–60 years (M = 3.89, SD = 0.99). Employees between 21–30 years showed significant differences (M = 2.35, SD = 0.98) towards well-being when compared with those 31–40 years (M = 2.08, SD = 0.87), 41–50 years (M = 1.91, SD = 0.88) and 51–60 years (M = 1.67, SD = 0.79). Finally, employees between 21–30 years showed significant differences (M = 3.99, SD = 0.98) towards life satisfaction when compared with those 41–50 years (M = 4.37, SD = 0.94). 

The highest education level of cruise ship employees varied; important differences were observed between the education level of employees and research model variables. The highest education level of employees had observable effects on social pressure (F(3, 141.54) = 3.08, p = 0.03), internet multitasking (F(3, 519) = 5.05, p = 0.00), perceived social support (F(3, 141.54) = 22.97, p = 0.00), well-being (F(3, 139.18) = 3.11, p = 0.03) and life satisfaction (F(3, 146.85) = 17.93, p = 0.00). Existing differences among education level and social pressure (η2 = 0.02), internet multitasking (η2 = 0.03), and well-being (η2 = 0.02) were low. However, differences among education level and life satisfaction (η2 = 0.07) and perceived social support (η2 = 0.05) were moderate. Interestingly, Student’s t-test revealed that employees with bachelor’s degrees showed significant differences (M = 1.15, SD = 0.35) towards internet multitasking when compared with employees with high school diplomas (M = 1.00, SD = 0.33) and master/doctoral degrees (M = 1.00, SD = 0.40). Employees with master/doctoral degrees show significant differences (M = 3.41, SD = 0.28) towards perceived social support when compared with employees with high school diplomas (M = 2.99, SD = 0.67), associates degrees (M = 1.91, SD = 0.88) and bachelor’s degrees (M = 3.06, SD = 0.49). Employees with associates degrees showed significant differences (M = 2.34, SD = 1.02) towards well-being when compared with employees with bachelor’s degrees (M = 2.05, SD = 0.83) and master/doctoral degrees (M = 1.87, SD = 1.05). Employees with master/doctoral degrees showed significant differences (M = 4.84, SD = 0.69) towards life satisfaction when compared with employees with high school diplomas (M = 3.94, SD = 1.12), associates degrees (M = 3.90, SD = 0.99) and bachelor’s degrees (M = 4.21, SD = 0.83). 

Cruise ship employees come from various countries around the globe. Thus, it is important to determine whether any fundamental differences were present based on country of residence and the research model variables. Employee place of residence had prominent effects on internet and online communication (F(6, 516) = 3.47, p = 0.00), fear of missing out (F(6, 516) = 2.91, p = 0.01), social pressure (F(6, 516) = 3.44, p = 0.00), relatedness to friends and family need satisfaction (F(6, 94.46) = 8.12, p = 0.00), internet multitasking (F(6, 92.36) = 4.34, p = 0.00), perceived social support (F(6, 87.66) = 3.95, p = 0.00) and well-being (F(6, 91.14) = 3.03, p = 0.01). Differences between place of residence and fear of missing out (η2 = 0.03) were low. Differences among place of residence and internet and online communication (η2 = 0.04), social pressure (η2 = 0.04), relatedness to friends and family need satisfaction (η2 = 0.04), internet multitasking (η2 = 0.04), well-being (η2 = 0.02) and perceived social support (η2 = 0.06) were moderate. Student’s t-test revealed that employees from Africa showed significant differences (M = 2.71, SD = 0.94) towards internet and online communication when compared with employees from Central America (M = 3,54, SD = 0.93) and South America (M = 3,61, SD = 0.77). Employees from South America showed significant differences (M = 4.26, SD = 0.90) towards fear of missing out when compared with employees from Europe (M = 3.71, SD = 0.91). Employees from South America showed significant differences (M = 3.12, SD = 0.65) towards social pressure when compared with employees from Europe (M = 2.78, SD = 0.65) and Australia (M = 2.54, SD = 0.84). Employees from South America showed significant differences (M = 4.49, SD = 0.27) towards relatedness to friends and family need satisfaction when compared with employees from Europe (M = 4.09, SD = 0.71), Southeast Asia (M = 4.10, SD = 0.69) and Australia (M = 3.92, SD = 0.91). Internet multitasking was another variable where place of residence showed an effect; noticeable differences were observed among employees from Europe (M = 4.09, SD = 0.71), Central America (M = 4.29, SD = 0.43), South America (M = 4.49, SD = 0.27) and Australia (M = 3.92, SD = 0.91). Employees from North America showed significant differences (M = 3.18, SD = 0.41) towards perceived social support when compared with employees from Southeast Asia (M = 2.92, SD = 0.51) and Australia (M = 2.58, SD = 0.83).

## 4. Discussion and Conclusions

The goal of the current study was to investigate and model complex mutual interactions the Internet and online communication had on social pressure, fear of missing out, internet multitasking and relatedness to friends and family need satisfaction, perceived social support, well-being and life satisfaction of cruise ship employees. The results revealed that the Internet and online communication have positive effects on social pressure and fear of missing out, whereas social pressure and fear of missing out have positive effects on relatedness to friends and family need satisfaction and internet multitasking. Moreover, internet multitasking had a positive effect on perceived social support, which in turn had a positive effect on life satisfaction. 

While onboard a cruise ship, employees are detached from their family, significant others and friends. Thus, employees experience strong social pressure to be constantly available and fear of missing out on important information and life events. Due to their rigorous schedule, i.e., working 10 to 13 hours every day of the week in an isolated environment, the Internet and online communications are wonderful instruments that can meet the demands of social pressure and reduce the fear of missing out experienced by cruise ship employees. The Internet and online communication are essential to employees from collectivistic cultures where friend and family ties are strong. Thus, providing these employees instruments to maintain close contacts with friends and family at home will reduce their social pressure and fear of missing out, which in turn will create harmony among employee social groups. Moreover, the provision of online communication creates an interactive platform for validation through communication acceptance; validation boosts employee sense of belonging and strengthens their relationships with friends and family at home. These results are supported by the Theory of Belongingness by [19] and are in line with previous studies conducted by [5,8,53,83]. 

Free time is the single most precious commodity for cruise ship employees. Tight work schedules, ship itineraries, in-port safety duties, and daily job demands leave employees with very narrow time windows for engagement in social networking sites and online communication. Thus, social pressure and fear of missing out effects on relatedness to friends and family need satisfaction force employees to engage in internet multitasking. Within the minimal and confined space of a cruise ship, employees lose almost all points of reference to the outside world and friends and family at home; thus, social pressure and fear of missing out fuels the need for relatedness to friends and family and internet multitasking. Employees spend on average between four and six months onboard in small shared cabins while working long hours every day of their full contract length and are in desperate need of the support of family and friends. Thus, if cruise ship companies provide ad libitum internet access to online social networks and communication, employees will enjoy a strong network of supportive family and friends that can help them enhance life satisfaction. Interestingly, employees with bachelor’s degrees exhibited high levels of internet multitasking, which correlated positively with their working memory and ability to divert their attention among different tasks. These results are supported by Self-Determination theory of [20] as well as Uses and Gratification Theory [21]. Moreover, these findings are in line with previous studies conducted by [11,13,18,33].

Cruise ship employees understand they are dispensable and replaceable to cruise ship companies [9]. Thus, relatedness to friends and family needs satisfaction is of paramount importance for employees because they know that to their friends and family, they are indispensable and unique. Moreover, living and working onboard a cruise ship is a lifestyle; while this particular lifestyle may seem to disregard certain employee rights, it does not dehumanize the employee. When employees feel their needs towards relatedness are satisfied, they experience elevated social support through strengthened connections with friends and family. Although cruise ship employees work and live on the high seas, they do not thrive alone: Donne [84] pointed out that each man relies on others. However, there are slight differences between employees and their relatedness to friends and family who need satisfaction. Experienced employees with 6+ years exhibited stronger needs towards relatedness because they feel lonely and isolated for a significant period of their life. Employees working in the entertainment department and employees coming from South America expressed greater needs because they have a wide circle of close ties at home. Lastly, the youngest cruise ship employees between 21–30 years exhibited greater needs towards relatedness because at their age, friendships are highly complex and offer significant self-disclosure and support. These results are also supported by Self-Determination Theory of [20] and are in line with previous studies conducted by [5,18].

Onboard cruise ship operations are in constant flux, and employees come and go frequently. For many employees these crew changes become the only point of reference. During long contracts, tiredness of employees builds up as physical pain, exhaustion and psychic fatigue. However, cruise ship employees are required to continuously work until the completion of their contracts. Experiences like these build highly intense relationships where employees need social support. In such an environment, employees lean on their friends and family at home for support, which comes in many forms most often as empathy, compassion and providing care. Social support is the foundation of healthy relationships that improves employee life satisfaction. Employees who spent 6+ years living and working on cruise ships are exposed to prolonged periods of loneliness and isolation, which affects them in ways that would require social support to achieve happiness and life satisfaction. Male employees look for social support to feel happy and satisfied, whereas female employees engage in social support to pursue increased well-being. Employees with master/doctoral degrees enjoy social support from their close ties because they understand the benefits of happiness that comes from such relationships. Finally, employees from North America consider deep relationships to have significance in enhancing social support because larger social networks improve life satisfaction. These results are supported by the positive psychology paradigm by [23]. Moreover, these findings are in line with previous studies conducted by [5,8,11]. 

Although keeping in touch with family and friends at home is essential for cruise ship employees, the majority of cruise companies charge significant prices for internet and online communication services. Thus, due to limitations of internet and online communication use because of high service prices, poor coverage and slow data connection, employees are prevented from satisfying their needs through their friends and family at home. Moreover, due to in-port manning duties, employees cannot use free internet services off ship, which leads to chronic emotional distress, frustration, anger, despair, and anxiety. This finding is in line with [25] who argued that people heavily dependent on social networking sites to satisfy their needs towards relatedness to friends and family may experience a lack of social capital outcomes; this can trigger detrimental impacts on their well-being. Employment on cruise ships carries many occupational safety hazards [4]. To compensate, employees engage in internet multitasking; however, doing so exposes employees to added distractions and prevents them from safely or effectively completing their tasks. Moreover, employees who engage in other tasks/activities while taking part in online communication with friends and family exhibit reduced performance, which leads to poor interaction and social dissatisfaction. This finding was in line with [56] who argued that multitasking is related to reduced enjoyment in messages and reduced recognition memory performance. Similarly, Örün et al. [55] argue that retention of communication content during online messaging is significantly worse while multitasking. Lastly, the perceived social support from internet and online communications and social networking sites with family and friends at home is dependent on reciprocity. Employees experiencing under-reciprocating exchanges show significant negative effects on their well-being. These results are supported by the Conservation of Resources Theory [22] and the reciprocity norm [85]. Perceived social support is a multi-dimensional construct highly dependent on personality traits. Personality can affect perceived social support relationship with well-being to the point of being non-significant [86]. Overall, our results provided several important theoretical and practical implications relevant to cruise tourism and human resource management.

### 4.1. Theoretical Implications

This study contributes to academic literature in several ways. First, working and living on a cruise ship, carries a heavy burden where cruise ship employees are detached from their family and friends at home [9] and their main communication instruments are the Internet, social networking sites and online communications [5]. Thus, although this study is of an exploratory and pioneering nature regarding the effects of internet communication on employee well-being and life satisfaction, this study was founded on well-known theories. Overall, the results are consistent with the Theory of Belongingness [19], the Self-Determination Theory [20], the Uses and Gratification Theory [21] and the Conservation of Resources Theory [22] and confirmed previous studies that reported positive effects of the Internet and online communication on social pressure on fear of missing out [5,11]; relatedness to friends and family needs satisfaction [18]; fear of missing out on internet multitasking [13]; relatedness to friends and family needs satisfaction and perceived social support [8]; and life satisfaction [5]. Intriguingly, the results did not confirm positive effects of internet and online communication on relatedness to friends and family needs satisfaction, internet multitasking on perceived social support, and perceived social support on well-being. However, these findings are supported by previous studies, such as [25,55,56,86]. 

A second contribution to the literature is the final model’s broad scope and applicability towards achieving life satisfaction of employees who work and live in a specific workplace, as described by [87]. Comprehensive measurement scales (based on previously confirmed scales by [13,75,76,77,78] for measuring internet and online communication effects on well-being and life satisfaction of cruise ship employees showed strong reliability and validity. These tools can be used in future studies as instruments for measuring internet and online communication effects on the well-being and life satisfaction of various employees.

### 4.2. Practical Implications

This study offers valuable practical recommendations for cruise ship companies. Unhappy, detached and dissatisfied employees can become unproductive and disengaged, which can lead to high employee turnover, absenteeism, and increased expenses due to health care costs and insurance premium fees [3]; this can harm the profitability of cruise ship companies. Companies that provide free-of-charge internet and access to social networking sites and online communication, will satisfy employee needs for belongingness. This will allow employees to enjoy a strong network of family and friends to achieve and maintain life satisfaction. Moreover, companies who understand and appreciate the value of employees who flourish in life satisfaction should reinforce their core values by setting their “true north" towards providing employees instruments to maintain close contacts with their friends and family at home. Such provisions by companies would suppress employee social pressure and fear of missing out. This would create harmony among their close ties and social groups, ultimately leading to life satisfaction of cruise ship employees.

### 4.3. Limitations and Future Research

This study has several limitations. First, this study utilized a cross-sectional time horizon; there is space for potential causality and reciprocal relationships among components [88]. Future studies should use a longitudinal time horizon to investigate the effects of the Internet and online communication on well-being and life satisfaction during various stages of cruise ship employee contracts. Second, common method bias is expected in this study due to self-reported answers collected from employees who agreed to participate in the survey. To lessen this challenge, a cautiously composed and validated survey was used following the suggestion of [89]. As such, participant anxiety related to giving right or wrong answers was at least reduced to its lowest possible level, if not completely avoided. Nevertheless, components that were used in this study could only be measured by particular, authentic impressions of employees. Third, participants in this study were recruited via the Facebook group “Crew Center”. Thus, the sample is not representative of the general population of all cruise ship employees who use the Internet and online communications because many cruise ship employees are not members of the aforementioned group and as such are underrepresented. The fourth limitation is related to the sampling method, i.e., the convenience sample method. This method could have limited the generalizability of the overall findings. The fifth limitation is related to the research model. Even though components showed satisfactory levels of validity and reliability, component constituents should be tested in future work on wider populations of employees. The sixth limitation is the quantitative analyses that were used to evaluate research data. Future studies could mix qualitative and quantitative techniques to obtain comprehensive knowledge about the effects of internet and online communication on well-being and life satisfaction of cruise ship employees. Taking into consideration these limitations, the results of this exploratory study cannot be generalized and should be judged with limited and careful interpretation. 

## Figures and Tables

**Table 1:** Sample characteristics.

Respondent Profile (n = 532)			
**Gender**	**Women**	195	37.3%
	Men	328	62.7%
**Age**	21–30	145	27.7%
	31–40	266	50.9%
	41–50	62	11.9%
	51–60	30	5.7%
	60+	20	3.8%
**Education**	High school	78	14.9%
	Associate degree	93	17.8%
	Bachelor’s degree	302	57.7%
	Master’s degree & Doctoral degree	50	9.6%
**Residence**	North America	106	20.3%
	Europe	229	43.8%
	Central America	24	4.6%
	Southeast Asia	86	16.4%
	South America	38	7.3%
	Africa	22	4.2%
	Australia	18	3.4%
**Department**	Hotel	259	49.5%
	Marine and Technical	172	32.9%
	Entertainment	92	17.6%
**Years of service**	1–2	106	20.3%
	3–5	177	33.8%
	6+	240	45.9%

**Table 2:** Test of hypothesis.

Hypothesis	CB-SEM		Conclusions
	Coefficient	T-Value	
Hypothesis 1: There is a positive relationship between the internet and online communication and social pressure.	0.237	4.185	Accept
Hypothesis 2: There is a positive relationship between the internet and online communication and fear of missing out	0.169	3.273	Accept
Hypothesis 3: There is a positive relationship between the internet and online communication and relatedness to friends and family need satisfaction.	0.075	1.532	Reject
Hypothesis 4: There is a positive relationship between social pressure and relatedness to friends and family need satisfaction.	0.358	6.401	Accept
Hypothesis 5: There is a positive relationship between fear of missing out and internet multitasking.	0.248	3.879	Accept
Hypothesis 6: There is a positive relationship between relatedness to friends and family need satisfaction and perceived social support.	0.148	2.599	Accept
Hypothesis 7: There is a positive relationship between internet multitasking and perceived social support.	−0.008	−0.146	Reject
Hypothesis 8: There is a positive relationship between perceived social support and well-being.	−0.127	−2.475	Reject
Hypothesis 9: There is a positive relationship between perceived social support and life satisfaction.	0.522	6.115	Accept

## Appendix A

**Table A1:** Scale items used for CB-SEM.

Item	Reference
***Internet and online communication***	Scale developed by authors based on Churchill’ principles (1979)
Usage of internet for communication throughout a week	
Average of hours per day spent on internet communication	
Usage of instant messenger throughout a week or day	
Usage of social networking sites throughout a week or day	
Usage of chat rooms throughout a week or day	
***Social pressure***	Reinecke, Aufenanger, Beutel, Dreier, Quiring, Stark, Wölfling, & Müller (2017)
People from private social environment that think it is important to be constantly available	
People from private social life that care and appreciate being constantly available	
Friends that expect being constantly available	
Feeling of social obligation to be constantly available	
***Fear of missing out***	Reinecke, Aufenanger, Beutel, Dreier, Quiring, Stark, Wölfling, & Müller (2017)
Less frequent usage of Internet would lead to a fear of missing out on important things	
Less frequent usage of Internet would lead to a fear of losing a track on friends and acquaintances	
Less frequent usage of Internet would lead to a fear of not being up-to-date anymore	
***Relatedness to friends and family need satisfaction***	Shen (2010)
Feeling that friends and/or family sincerely care about	
Feeling that friends and/or family honestly enjoys their spent time	
Feeling that friends and/or family seems genuinely interested about	
***Internet multitasking***	Reinecke, Aufenanger, Beutel, Dreier, Quiring, Stark, Wölfling, & Müller (2017)
Frequency of using internet simultaneously while using other media	
Frequency of using internet simultaneously while in conversation with another person	
Frequency of using internet simultaneously while in a meal with another person	
Frequency of using internet simultaneously while being in interaction with romantic partner	
Frequency of using internet simultaneously while being out with friends	
***Perceived social support***	Zimet, Dahlem, Zimet, & Farley (1988)
A special person who is around when needed	
Emotional help and support from family	
Support from friends when things go wrong	
Conversation about problems with family members	
Friends with whom joys and sorrows can be shared	
A special person in life who cares	
***Life satisfaction***	Diener, Emmons, Larsen, & Griffin, S. (1985)
A life is close to the ideal one	
Life conditions are excellent	
Overall satisfaction with life	
Having the important things in life	
If life could be lived over again, almost nothing would be changed	
***Well-being***	World Health Organization (1998)
Cheerful and good spirit feelings	
Feelings of calamity and relaxation	
Active and vigorous feelings	
Feelings of being fresh and rested	
Daily life being filled with things of personal interest	
